# TERT genetic polymorphism *rs2736100* is associated with an aggressive manifestation of papillary thyroid carcinoma

**DOI:** 10.3389/fsurg.2022.1019180

**Published:** 2023-01-10

**Authors:** Xilin Nie, Jinbiao Shang, Wendong Wang

**Affiliations:** Department of Thyroid Surgery, Institute of Cancer Research and Basic Medical Sciences of Chinese Academy of Sciences, Cancer Hospital of University of Chinese Academy of Sciences, Zhejiang Cancer Hospital, Hangzhou, China

**Keywords:** papillary thyroid carcinoma (PTC), TERT, *rs2736100*, manifestation, polymorphism

## Abstract

**Objectives:**

*TERT rs2736100* genetic polymorphism is commonly found in human malignancies, indicating its key role in cancer cell transformation. The aim of this study is to investigate the effects of the functional *TERT rs2736100* genetic polymorphism on the outcomes of papillary thyroid carcinoma (PTC) patients.

**Materials and methods:**

We performed a retrospective study on the relationship between *rs2736100* and clinicopathological outcomes of PTC in 500 patients (378 females and 122 males) aged 43.8 ± 11.4 years (range 15–74 years) with a median follow-up of 60 months (range, 1–455 months).

**Results:**

*TERT rs2736100* genetic polymorphism (TG/GG vs. TT) was significantly associated with several high-risk clinicopathological features such as tumor spread, extrathyroidal extension, central/lateral lymph node metastases, and Stage T III or IV disease. However, in Kaplan–Meier survival analyses, the *rs2736100* mutation was unrelated to overall disease-free survival with a log-rank value of *p* > 0.05. In Cox-regression analyses, the overall survival rate of recurrence/neo-metastasis was related to a larger tumor size, younger age, and tumor spread but unrelated to the *rs2736100* mutation.

**Conclusions and significance:**

*TERT rs2736100* genetic polymorphism mutation is more likely to manifest with aggressive clinicopathological characteristics but cannot worsen prognosis in PTC.

## Introduction

The rate of incidence of thyroid carcinoma increases by 4% per year, of which papillary thyroid carcinoma (PTC) accounts for approximately 80% and for 1% for all human malignancies (1). All thyroid malignancies mostly metastasize to the cervical lymph nodes, with 18%–90% of patients developing metastasis in the cervical region, which is associated with a higher rate of loco-regional recurrence and distant metastasis (2–4). Despite this high metastasis rate, PTC still has an excellent prognosis as an indolent disease for a majority of people (5). However, cervical lymph node metastasis is very common in PTC and is associated with an increased risk of loco-regional recurrence and overall mortality in a selected patient population (6–8). As a result, controlling loco-regional recurrence has become a major challenge for most thyroid surgeons (9).

Accumulated evidence demonstrates that the ch 5p15.33 region (*TERT-CLPTM1L*) is a common susceptibility locus of multiple cancers. Genome-wide association studies (GWAS) declare that many independent susceptibility single-nucleotide polymorphisms (SNPs) in 5p15.33 are identified in different malignancies such as lung cancer (10, 11), melanoma and non-melanoma skin cancer (12) and so on. Therefore, it is plausible that several functional DNA elements might exist in this region and influence cancer etiology. There are two known oncogenes, *TERT* and *CLPTM1L*, in this locus. Activated *TERT* (telomerase reverse transcriptase) transcription enhances telomerase activities and accelerates malignant transformation (13, 14). In lung cancer, oncogene *CLPTM1L* (cleft lip and palate-associated transmembrane 1 like protein) plays a pro-tumorigenic role and is critical for RAS-driven lung cancers (15, 16). In pancreatic cancer, *CLPTM1L* functions as a growth-promoting gene and its overexpression might lead to an abrogation of normal cytokinesis and enhance aneuploidy in pancreatic cancer cells (17).

However, the involvement of this locus in the etiology of PTC is still largely unknown. Here, we systematically evaluate PTC-susceptible genetic variants in the *TERT-CLPTM1L* locus and their regulatory role in *TERT* gene expression *ex vivo* and *in vivo*. *Ex vivo* luciferase gene assays demonstrate that the PTC-susceptible *rs2736100* polymorphism locates in a potential *TERT* intronic enhancer and has a genotype-specific impact on *TERT* expression. In addition, the correlation between the *rs2736100* genotype and the tissue-specific *TERT* gene expression level supports the regulatory function of this genetic variant *in vivo* (18).

Based on our experiment results, the functional *TERT* rs2736100 genetic polymorphism may be deemed as a novel genetic component of PTC etiology in Chinese populations. Now, a question arises. In neoplasia development, what is the effect of *TERT rs2736100* genetic polymorphism on clinical progress? In this research, we retrospectively analyze the clinicopathological features of PTC patients in our hospital in order to determine the effects of the functional *TERT rs2736100* genetic polymorphism on the outcomes of these patients.

## Materials and methods

In the last report, the clinicopathological features and outcomes of all patients were retrieved from medical records and were analyzed to explore the relationship between the *TERT rs2736100* genetic polymorphism mutation and no mutation. These patients were treated for PTC or clinically observed between October 2013 and December 2013 at Zhejiang Cancer Hospital. This study included 500 patients (378 females and 122 males) aged 43.8 ± 11.4 years (mean ± SD, range 15–74 years, age of initial diagnosis) with a median follow-up of 60 months (range, 1–455 months) after initial surgery. There were 20 patients coexisting with other malignancies such as breast carcinomas (4), nasopharyngeal carcinomas (10), and esophagus carcinomas (6), which were excluded in the GWAS. At the same time, some of these patients had received external radiation therapy in the neck, including the thyroid gland, before undergoing thyroid surgery, which was also excluded in the GWAS. All subjects gave written consent and the study was approved by the Regional Ethical Committee of Zhejiang Cancer Hospital in accordance with the Helsinki Declaration.

The clinicopathological features were retrospectively analyzed, such as gender, age (≤45 or >45 years), tumor size (≤1 or >1 cm), tumor spread, tumor multifocality, extrathyroidal extension (ETE), central lymph node metastases (CLNM), lateral lymph node metastases (LLNM), pTNM stage, recurring, and/or distant metastasis. When multiple lesions were found in the specimen, the largest tumor or the most suspicious nodule was analyzed. The TNM stage of the tumor is based on the American Joint Committee on Cancer (AJCC)/Union for International Cancer Control (UICC) classification system (19).

### Statistical analysis

Categorical data were summarized with frequencies and percentages. Continuous data were summarized with means ± standard deviations (if normally distributed) or medians and interquartile ranges (if not normally distributed). Comparisons of categorical variables were performed using the *χ*^2^ test or Fisher's exact test for small cell sizes. The independent *t*-test and Wilcoxon–Mann–Whitney tests were used for normally and non-normally distributed continuous variables, respectively. We used Kaplan–Meier survival curves with the long-rank test to present either overall survival (considering only disease-related deaths) or disease-free survival (where “disease” was defined as persistent or recurrent lymph nodal or distant metastases, or disease-related death). The associations between TERT and PTC risk were estimated by odds ratios (ORs) and their 95% confidence intervals (CIs) computed by logistic regression models. All ORs were adjusted for age or sex, where it was appropriate. Follow-up time was defined as the time interval from the initial thyroidectomy to the discovery of disease recurrence and/or distant metastases. If there was no recurrence, the time of the last follow-up visit (around October 2016) interval was used to calculate recurrence-free survival rates by mutation status. Independent associations of mutations with PTC recurrence were examined by Cox-regression analyses. All *p-*values had two sides, and a *p-*value of <0.05 was considered statistically significant. The analyses were performed using Statistical Package for Social Sciences (SPSS, Inc., Chicago, IL, United States).

## Results

### *TERT rs2736100* genetic polymorphism in PTC

The study cohort was described previously (18). The *rs2736100* polymorphism variant distribution (TT 136, TG 238, and GG 126) is shown in Table 1. The diameter of the tumor ranged from 0.1 to 7.2 cm with a median diameter of 1.5 cm. Among all patients, 163 patients showed tumor spread in the thyroid gland, 325 had more than one lesion, and 226 patients had ETE. As many as 346 patients had central lymph node metastases and 196 patients had lateral lymph node metastases, and the number of positive lymph nodes ranged from 1 to 21. In addition, 14 patients had metastases in distant sites such as the lungs or bone at the initial diagnosis. Overall, 111 patients experienced recurrence of lymph nodes at the central or lateral compartment of the neck and 39 patients experienced distant metastasis in the lungs or bone and in other sites*.* (14 patients with metastases at the initial diagnosis were not included, simplified as neo-distant metastasis), and only one patient had metastases in the brain. There were 363 disease-free patients at the end of the follow-up period. Only two patients died of PTC, one with a tumor size of 7.2 cm who refused any treatment for tracheal constriction and apnea, and the other with local advanced disease (larynx, trachea, and esophagus invasion) and untreated. The baseline characteristics of the cohort according to the presence or absence of *TERT rs2736100* genetic polymorphism mutations are presented in Table 1.

**Table 1:** Demographic clinicopathological features of 500 patients.

Clinicopathological features		*rs2736100* wild	*rs2736100* mutation			Univariate analysis *p*-value
		TT	TG	GG	Total	
Total no. of cases		136	238	126	364	
Gender	Female	98	179	101	280	0.260
** **	Male	38	59	25	84	
Age at diagnosis, years	M ± SD	43.2 ± 11.9	44.1 ± 11.2	43.8 ± 11.4	44.0 ± 11.2	0.492
** **	≤45	68	129	68	197	0.411
** **	>45	68	109	58	167	
Tumor size, cm	M ± SD	1.5 ± 1.0	1.6 ± 1.1	1.7 ± 1.3	1.6 ± 1.2	0.218
** **	≤1	63	104	52	156	0.487
** **	>1	73	134	74	208	
Tumor spread	Absent	101	157	79	236	**0**.045*
** **	Present	35	81	47	128	
Tumor multifocality	Single	55	76	44	120	0.119
** **	Multi	81	162	82	244	
ETE	Absent	89	137	48	185	**0**.002*
** **	Present	47	101	78	179	
CLNM	Absent	52	71	31	102	**0**.028*
** **	Present	84	167	95	262	
LLNM	Absent	95	162	47	209	**0**.011*
** **	Present	41	76	79	155	
pT	1	85	132	66	210	**0**.029*
** **	2	8	7	5		
** **	3	32	73	37	154	
** **	4	11	26	18		
pN	0	47	62	28	90	**0**.028*
** **	1	89	176	98	274	
pM	Absent	134	231	121	352	0.370
** **	Present	2	7	5	12	
Recurrence	Absent	104	189	96	285	0.662
** **	Present	32	49	30	79	
Neo-distant metastasis	Absent	127	212	111	323	0.257
** **	Present	7	19	10	29	
End-point event	Absent	99	175	89	264	0.953
** **	Present	37	63	37	100	

The bold values means statistical difference (*p* < =0.05).

* *p* < 0.05.

ETE, extrathyroidal extension; CLNM, central lymph node metastases; LLNM, lateral lymph node metastasis; pT, pathological tumor stage; pN, pathological lymph node metastasis stage; pM, pathological distant metastasis stage.

In the univariate analyses, the *rs2736100* mutations TG and GG were combined as mutation-positive like in the last report. The four pathological T stages were recategorized as I + II and III + IV. The overall analysis was found to be significantly associated with several high-risk clinicopathologic features, including tumor spread, ETE, CLNM, LLNM, and pathological T stages.

The others did not demonstrate any significant associations such as gender, age, tumor size, tumor multifocality, distant metastasis at diagnosis, recurrence, and neo-distant metastasis (*p* > 0.05).

In the multivariate analyses for *TERT rs2736100* genetic polymorphism, ETE was the only risk factor related to this type of polymorphism [*p* = 0.004, OR = 1.832, 95% CI: 1.217–2.757] (Table 2).

**Table 2:** Factors associated with the presence of *TERT rs2736100* genetic polymorphism (TT vs. TG/GG) in multivariate analysis.

Items	Risk factors	*p*	OR	95% CI	
				Lower	Upper
*rs2736100* mutation	ETE (present vs. absent)	**0.004** *	1.832	1.217	2.757

The bold values means statistical difference (*p*<=0.05).

* *p* < 0.05.

ETE, extrathyroidal extension; OR, odds ratio; CI, confidence interval.

### Relationship of *TERT rs2736100* genetic polymorphism with the clinicopathological outcomes of PTC

Tumor recurrence rate on lymph nodes was 78 of 285 in mutation-positive patients vs. 32 of 104 in mutation-negative patients. The recurrence was related to large size (*p* = 0.000, OR = 2.426, 95% CI: 1.520–3.871) and younger age (*p* = 0.008, OR = 0.544, 95% CI: 0.348–0.850 for Age > 45 years) after logistic regression for multivariate analysis. In the analysis for distant metastasis, 14 patients were excluded because of distant metastasis at initial diagnosis. The rate of tumor neo-distant metastases was 29 of 323 in mutation-positive patients vs. 7 of 127 in mutation-negative patients, and the metastases were related to large size only after logistic regression (*p* = 0.001, OR = 4.375, 95% CI: 1.786–10.716). The overall recurrence rate (the recurrence of lymph node and neo-distant metastasis was combined as an end-point event) was 100 of 264 in mutation-positive patients vs. 37 of 99 in mutation-negative patients. This rated was related to large size (*p* = 0.000, OR = 3.039, 95% CI: 1.955–4.724) and younger age (*p* = 0.046, OR = 0.656, 95% CI: 0.434–0.992 for Age > 45 years) after logistic regression. However, in these analyses, the *TERT rs2736100* mutation was unrelated to the recurrence, neo-distant metastasis, and end-point event (Table 3).

**Table 3:** Multivariate analyses for recurrence and/or neo-distant metastasis.

Items	Risk factors	*p*	OR	95% CI	
				Lower	Upper
Recurrence	Age (>45 vs. ≤45 years)	**0.008** *	0.544	0.348	0.850
	Size (>1 vs. ≤1 cm)	**0**.000*	2.426	1.520	3.871
Neo-distant metastasis	Size (>1 cm vs. ≤1 cm)	**0**.001*	4.375	1.786	10.716
End-point event	Age (>45 years vs. ≤45 years)	**0**.046*	0.656	0.434	0.992
	SIZE (>1 cm vs. ≤1 cm)	**0**.000*	3.039	1.955	4.724

The bold values means statistical difference (*p*<=0.05).

* *p* < 0.05.

OR, odds ratio; CI, confidence interval.

**Figure 1:**
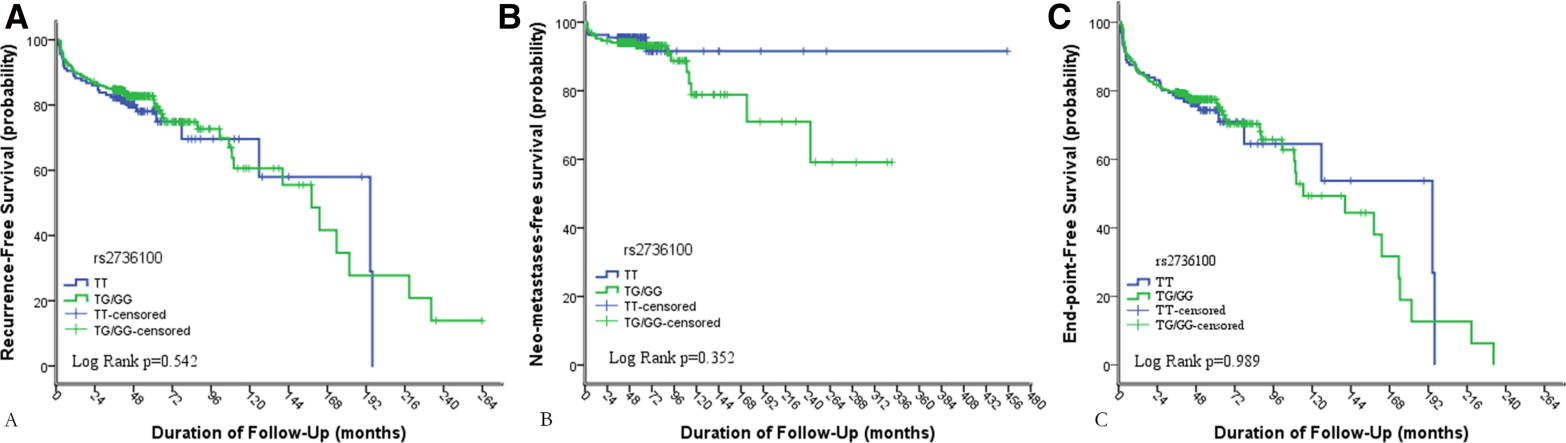
Results of Kaplan–Meier analyses of the impacts of *TERT rs2736100* genetic polymorphism on (**A**) the recurrence-free survival of patients with PTC; (**B**) the neo-metastasis-free survival rates of patients with PTC, 14 patients were excluded because of having distant metastasis at initial diagnosis; (**C**) the overall recurrence-free (the recurrence of lymph nodes and neo-distant metastases was combined as an end-point event) survival rates of PTC.

We performed Kaplan–Meier and log-rank analyses for calculating the disease-free survival rates of patients by genotype. In the analyses of all PTC patients, the overall recurrence and metastasis-free probability was unrelated to *TERT rs2736100* genetic polymorphism with log-rank analysis *p* = 0.542 and *p* = 0.242, respectively (*p* = 0.989 for combined) (Figures 1A–C).

In the Cox-regression analysis, the overall survival rate of recurrence was related to the larger size of the tumor [*p* = 0.002, risk ratio (RR) = 1.983, 95% CI: 1.296–3.034], younger age (*p* = 0.050, OR = 0.671, 95% CI: 0.451–1.000 for Age > 45 years), and tumor spread (*p* = 0.023, RR = 1.582, 95% CI: 1.064–2.352) and unrelated to *rs2736100* mutation and the others (Table 4).

**Table 4:** Cox-regression analysis result of the overall recurrence (the recurrence of lymph nodes and neo-distant metastases was combined).

Items	Risk factors	*p*	RR	95% CI	
				Lower	Upper
End-point event	Age (>45 vs. ≤45 years)	**0.050**	0.671	0.451	1.000
	Size (>1 vs. ≤1 cm)	**0**.002*	1.983	1.296	3.034
	Tumor spread (present vs. absent)	**0**.023*	1.582	1.064	2.352

The bold values means statistical difference (*p* < =0.05).

* *p* < 0.05.

RR, risk ratio; CI, confidence interval.

## Discussion

*TERT* is the catalytic subunit of telomerase, which plays an important role in cell immortalization and tumorigenesis. The two *TERT* promoter mutations were shown to be mutually exclusive and able to increase *TERT* expression (20, 21). They were also shown to be associated with the aggressiveness of other human cancers such as melanoma, brain tumor, bladder cancer, and papillary thyroid carcinoma (22–26). The prevalence of *TERT* promoter mutations in PTC varied between 7.5% and 27% in previous studies (26–33). In our last report on *rs2736100*, the *TERT rs2736100* genetic variant (TG/GG vs. TT) was significantly associated with elevated PTC risk. Correlations between *rs2736100* genotypes and tissue-specific *TERT* expression supported the regulatory function of this genetic variant *in vivo*. Our data demonstrated that the functional *TERT rs2736100* SNP was a novel genetic component of PTC etiology (18). However, whether genetic variants of *TERT-CLPTM1L* are associated with an increased risk of PTC in clinical outcomes is unknown.

Some studies showed an association between *BRAF V600E* and *TERT* promoter mutations. In the study by Xing et al. the coexistence of the two mutations was associated with the worst clinicopathological outcomes of PTC (29). *TERT C228T* alone was significantly associated with lymph node metastasis, and there was an insignificant association with other clinicopathological characteristics. In contrast, the coexistence of *BRAF V600E* and *TERT C228T* was strongly associated with virtually all high-risk characteristics as well as distant metastasis recurrence. In survival analysis, similar results demonstrated that *TERT* mutation alone cannot affect disease-free survival rates more than the coexistence of *BRAF V600E* and *TERT C228T*. Similar results were demonstrated in several reports; the coexistence of *BRAF V600E* and *TERT* promoter mutations was particularly associated with high-risk clinicopathological features (28, 34–38). These reports that implied the effects of *TERT C228T* mutation had reduced significance when they were separated from the *BRAF* mutation and examined alone, suggesting that the *TERT* mutation needs additional genetic alterations to promote an aggressive manifestation of PTC. However, in reports of Melo et al. that detected an association between *TERT* mutations and aggressive clinicopathological features, there was enough evidence to state that *TERT* promoter mutations with or without *BRAF V600E* was a major indicator of poor prognosis in differentiated thyroid cancer, and notably in PTC, due to its association with distant metastasis and increased disease-specific mortality (26, 39, 40). Unfortunately, the *BRAF* status was not assessed in this research. In addition, the coexistence of *TERT* rs2736100 mutations with/without *BRAF V600E* is particularly associated with some high-risk clinicopathological features but unassociated with prognosis on PTC patients in this research.

Moreover, the overall recurrence rate (the recurrence of lymph nodes and neo-distant metastases was combined as an end-point event) was 100 of 264 in mutation-positive patients vs. 37 of 99 in mutation-negative patients. The recurrence was related to large size (*p* = 0.000, OR = 3.039, 95% CI: 1.955–4.724) and younger age (*p* = 0.046, OR = 0.656, 95% CI: 0.434–0.992 for Age > 45 years) after logistic regression. However, TERT *rs2736100* genetic polymorphism was unrelated to recurrence, neo-distant metastasis, and end-point event in this study.

There were several reports that focused on *SNP re2736100*. In the study by Liu et al. only *rs2736100* was significantly (*p* = 0.034) associated with an increased risk of lung cancer and suggested that *rs2736100* on *TERT-CLPTM1L* indicates a poor prognosis for lung cancer in the Chinese Han population (41). In the report of Bae et al. SNPs at 5p15 (*rs2736100*, adjusted odds ratio 1.32, and 95% CI: 1.03–1.67, *p* = 0.025) were significantly associated with lung cancer risk (42). The report of Simon et al. indicated that *SNP rs2736100* risk genotypes were highly correlated with high-grade disease (*p* < 0.001), but *rs2736100* was unrelated to the prognosis of tumor grade in glioma independently (43). In the study by Choi et al. there was no significant difference between patients with gastric cancer and healthy controls in the genotype and allele frequencies of *rs2736100* polymorphism. The *rs2736100* polymorphism of the *hTERT* gene was involved in the regulation of *hTERT* expression and telomere length but not in the risk of gastric cancer (44). In the report by Chen et al. for *rs2736100*, the G variant and the GG genotype were more frequent, whereas the TT genotype was less frequent in patients with lung adenocarcinoma than in controls. They suggested that multiple variants at 5p15.33 contribute to susceptibility to lung adenocarcinoma (45). In these reports, only the report of Liu et al. demonstrated that *SNP re2736100* was significantly related to poor prognosis, with the others showing that *SNP rs2736100* was related only to several high-risk clinicopathological features excluding poor prognosis. Our results agree well with those reports that suggested that the overall recurrence-free (the recurrence of lymph nodes and neo-distant metastases was combined as an end-point event) survival rates of patients with PTC were unaffected significantly.

In our recent study, we observed that the *TERT rs2736100* mutation (either TG or GG) was significantly associated with some high-risk clinicopathological features such as tumor spread, extrathyroidal extension, central/lateral lymph node metastases, and Stage T III and IV disease. In multivariate analysis, the *TERT rs2736100* mutation was significantly associated only with extrathyroidal extension (*p* = 0.004, OR = 1.832, 95% CI: 1.217–2.757). However, in the Kaplan–Meier survival analysis, the *rs2376100* mutation was unrelated to the overall disease-free survival rate with a log-rank value of *p* > 0.05. In the Cox-regression analysis, the overall survival rate of recurrence was related to the larger tumor size (*p* = 0.002, RR = 1.983, 95% CI: 1.296–3.034), younger age (*p* = 0.050, OR = 0.671, 95% CI: 0.451–1.000 for Age > 45 years), and tumor spread (*p* = 0.023, RR = 1.582, 95% CI: 1.064–2.352) and unrelated to the *rs2376100* mutation and others.

The *TERT* mutation results are similar to those of the study by Myung et al. (46). No significant difference in the frequency of the TERT promoter mutation was observed between the recurrence/metastasis group and the non-recurrence/metastasis group. These results suggest that the prognostic implications of the *TERT* promoter mutation are dependent on clinicopathological features. In addition, in the report by Gong et al. *hTERT* gene polymorphism at *rs1006969C/T* is associated with the risk and prognosis of thyroid cancer but *hTERT* gene polymorphism at *rs2736100G/T* is not (47). Our results agree well with those of these reports.

The papillary thyroid carcinoma histotypes carry, in general, a good or even an excellent prognosis (5). *TERT rs2736100* mutation-positive PTC is more likely to manifest with aggressive clinicopathological characteristics. In appropriate clinical settings, testing for *TERT rs2736100* mutation-positive PTC is likely to be useful in assisting the risk stratification and management of PTC but not in predicting prognosis in clinical practices (48). These recent findings on *TERT rs2736100* mutation-positive PTC are exciting, but they remain to be confirmed and generalized by further and high-power studies, ideally in different ethnic populations.

In a recent study, we observed that the *TERT rs2736100* mutation (either TG or GG) was significantly associated with some high-risk clinicopathological features such as tumor spread, extrathyroidal extension, central/lateral lymph node metastases, and Stage T III and IV disease. However, in Kaplan–Meier survival analysis, the *rs2376100* mutation was unrelated to the overall disease-free survival with a log-rank value of *p* > 0.05. In the Cox-regression analysis, the overall survival rate of recurrence was related to the larger size of tumor (*p* = 0.002, RR = 1.983, 95% CI: 1.296–3.034), younger age (*p* = 0.050, RR = 0.671, 95% CI: 0.451–1.000 for Age > 45 years), and tumor spread (*p* = 0.023, RR = 1.582, 95% CI: 1.064–2.352) and unrelated to the *rs2376100* mutation and others. In addition, they remain to be confirmed and generalized by further and high-power studies, ideally in different ethnic populations.

## Data Availability

The original contributions presented in the study are included in the article/Supplementary Material, further inquiries can be directed to the corresponding author.

## References

[1] Shaha AR. Prognostic factors in papillary thyroid carcinoma and implications of large nodal metastasis. Surgery. (2004) 135(2):237–9. 10.1016/j.surg.2003.08.023 14739864

[2] Machens A Hofmann C Hauptmann S Dralle H. Locoregional recurrence and death from medullary thyroid carcinoma in a contemporaneous series: 5-year results. Eur J Endocrinol. (2007) 157(1):85–93. 10.1530/EJE-07-0095 17609406

[3] Sivanandan R Soo KC. Pattern of cervical lymph node metastases from papillary carcinoma of the thyroid. Br J Surg. (2001) 88(9):1241–4. 10.1046/j.0007-1323.2001.01843.x 11531874

[4] Kupferman ME Patterson M Mandel SJ LiVolsi V Weber RS. Patterns of lateral neck metastasis in papillary thyroid carcinoma. Arch Otolaryngol Head Neck Surg. (2004) 130(7):857–60. 10.1001/archotol.130.7.857 15262763

[5] Hunt JP Buchmann LO Wang L Abraham D. An analysis of factors predicting lateral cervical nodal metastases in papillary carcinoma of the thyroid. Arch Otolaryngol Head Neck Surg. (2011) 137(11):1141–5. 10.1001/archoto.2011.174 22106241

[6] Lundgren CI Hall P Dickman PW Zedenius J. Clinically significant prognostic factors for differentiated thyroid carcinoma: a population-based, nested case-control study. Cancer. (2006) 106(3):524–31. 10.1002/cncr.21653 16369995

[7] McConahey WM Hay ID Woolner LB van Heerden JA Taylor WF. Papillary thyroid cancer treated at the Mayo Clinic, 1946 through 1970: initial manifestations, pathologic findings, therapy, and outcome. Mayo Clin Proc. (1986) 61(12):978–96. 10.1016/S0025-6196(12)62641-X 3773569

[8] Hughes CJ Shaha AR Shah JP Loree TR. Impact of lymph node metastasis in differentiated carcinoma of the thyroid: a matched-pair analysis. Head Neck. (1996) 18(2):127–32. 10.1002/(SICI)1097-0347(199603/04)18:2<127::AID-HED3>3.0.CO;2-3 8647677

[9] Roh JL Park JY Rha KS Park CI. Is central neck dissection necessary for the treatment of lateral cervical nodal recurrence of papillary thyroid carcinoma? Head Neck. (2007) 29(10):901–6. 10.1002/hed.20606 17405173

[10] Rafnar T Sulem P Stacey SN Geller F Gudmundsson J Sigurdsson A Sequence variants at the TERT-CLPTM1L locus associate with many cancer types. Nat Genet. (2009) 41(2):221–7. 10.1038/ng.296 19151717 PMC4525478

[11] Landi MT Chatterjee N Yu K Goldin LR Goldstein AM Rotunno M A genome-wide association study of lung cancer identifies a region of chromosome 5p15 associated with risk for adenocarcinoma. Am J Hum Genet. (2009) 85(5):679–91. 10.1016/j.ajhg.2009.09.012 19836008 PMC2775843

[12] Yang X Yang B Li B Liu Y. Association between TERT-CLPTM1L rs401681[C] allele and NMSC cancer risk: a meta-analysis including 45,184 subjects. Arch Dermatol Res. (2013) 305(1):49–52. 10.1007/s00403-012-1275-8 22893025

[13] Alzahrani AS Alsaadi R Murugan AK Sadiq BB. TERT promoter mutations in thyroid cancer. Horm Cancer. (2016) 7(3):165–77. 10.1007/s12672-016-0256-3 26902827 PMC10355936

[14] Heidenreich B Rachakonda PS Hemminki K Kumar R. TERT promoter mutations in cancer development. Curr Opin Genet Dev. (2014) 24:30–7. 10.1016/j.gde.2013.11.005 24657534

[15] James MA Vikis HG Tate E Rymaszewski AL You M. CRR9/CLPTM1L regulates cell survival signaling and is required for Ras transformation and lung tumorigenesis. Cancer Res. (2014) 74(4):1116–27. 10.1158/0008-5472.CAN-13-1617 24366883 PMC6005686

[16] James MA Wen W Wang Y Byers LA Heymach JV Coombes KR. Functional characterization of CLPTM1L as a lung cancer risk candidate gene in the 5p15.33 locus. PLoS One. (2012) 7(6):e36116. 10.1371/journal.pone.0036116 22675468 PMC3366984

[17] Jia J Bosley AD Thompson A Hoskins JW Cheuk A Collins I CLPTM1L promotes growth and enhances aneuploidy in pancreatic cancer cells. Cancer Res. (2014) 74(10):2785–95. 10.1158/0008-5472.CAN-13-3176 24648346 PMC4030677

[18] Ge M Shi M An C Yang W Nie X Zhang J Functional evaluation of TERT-CLPTM1L genetic variants associated with susceptibility of papillary thyroid carcinoma. Sci Rep. (2016) 6:26037. 10.1038/srep26037 27185198 PMC4869017

[19] Edge SB Compton CC. The American Joint Committee on Cancer: the 7th edition of the AJCC cancer staging manual and the future of TNM. Ann Surg Oncol. (2010) 17(6):1471–4. 10.1245/s10434-010-0985-4 20180029

[20] Huang FW Hodis E Xu MJ Kryukov GV Chin L Garraway LA. Highly recurrent TERT promoter mutations in human melanoma. Science. (2013) 339(6122):957–9. 10.1126/science.1229259 23348506 PMC4423787

[21] Horn S Figl A Rachakonda PS Fischer C Sucker A Gast A TERT promoter mutations in familial and sporadic melanoma. Science. (2013) 339(6122):959–61. 10.1126/science.1230062 23348503

[22] Killela PJ Reitman ZJ Jiao Y Bettegowda C Agrawal N Diaz LA Jr TERT promoter mutations occur frequently in gliomas and a subset of tumors derived from cells with low rates of self-renewal. Proc Natl Acad Sci U S A. (2013) 110(15):6021–6. 10.1073/pnas.1303607110 23530248 PMC3625331

[23] Rachakonda PS Hosen I de Verdier PJ Fallah M Heidenreich B Ryk C TERT promoter mutations in bladder cancer affect patient survival and disease recurrence through modification by a common polymorphism. Proc Natl Acad Sci U S A. (2013) 110(43):17426–31. 10.1073/pnas.1310522110 24101484 PMC3808633

[24] Griewank KG Murali R Puig-Butille JA Schilling B Livingstone E Potrony M TERT Promoter mutation status as an independent prognostic factor in cutaneous melanoma. J Natl Cancer Inst. (2014) 106(9):dju246. 10.1093/jnci/dju246 25217772 PMC4200061

[25] Gandolfi G Ragazzi M Frasoldati A Piana S Ciarrocchi A Sancisi V. TERT promoter mutations are associated with distant metastases in papillary thyroid carcinoma. Eur J Endocrinol. (2015) 172(4):403–13. 10.1530/EJE-14-0837 25583906

[26] Melo M da Rocha AG Vinagre J Batista R Peixoto J Tavares C TERT promoter mutations are a major indicator of poor outcome in differentiated thyroid carcinomas. J Clin Endocrinol Metab. (2014) 99(5):E754–65. 10.1210/jc.2013-3734 24476079 PMC4191548

[27] Liu T Wang N Cao J Sofiadis A Dinets A Zedenius J The age- and shorter telomere-dependent TERT promoter mutation in follicular thyroid cell-derived carcinomas. Oncogene. (2014) 33(42):4978–84. 10.1038/onc.2013.446 24141777

[28] Liu X Qu S Liu R Sheng C Shi X Zhu G TERT promoter mutations and their association with BRAF V600E mutation and aggressive clinicopathological characteristics of thyroid cancer. J Clin Endocrinol Metab. (2014) 99(6):E1130–6. 10.1210/jc.2013-4048 24617711 PMC4037723

[29] Xing M Liu R Liu X Murugan AK Zhu G Zeiger MA BRAF V600e and TERT promoter mutations cooperatively identify the most aggressive papillary thyroid cancer with highest recurrence. Am J Clin Oncol. (2014) 32(25):2718–26. 10.1200/JCO.2014.55.5094 PMC4145183 25024077

[30] Muzza M Colombo C Rossi S Tosi D Cirello V Perrino M Telomerase in differentiated thyroid cancer: promoter mutations, expression and localization. Mol Cell Endocrinol. (2015) 399:288–95. 10.1016/j.mce.2014.10.019 25448848

[31] Liu X Bishop J Shan Y Pai S Liu D Murugan AK Highly prevalent TERT promoter mutations in aggressive thyroid cancers. Endocr Relat Cancer. (2013) 20(4):603–10. 10.1530/ERC-13-0210 23766237 PMC3782569

[32] Landa I Ganly I Chan TA Mitsutake N Matsuse M Ibrahimpasic T Frequent somatic TERT promoter mutations in thyroid cancer: higher prevalence in advanced forms of the disease. J Clin Endocrinol Metab. (2013) 98(9):E1562–6. 10.1210/jc.2013-2383 23833040 PMC3763971

[33] Vinagre J Almeida A Populo H Batista R Lyra J Pinto V Frequency of TERT promoter mutations in human cancers. Nat Commun. (2013) 4:2185. 10.1038/ncomms3185 23887589

[34] Jin L Chen E Dong S Cai Y Zhang X Zhou Y BRAF and TERT promoter mutations in the aggressiveness of papillary thyroid carcinoma: a study of 653 patients. Oncotarget. (2016) 7(14):18346–55. 10.18632/oncotarget.7811 26943032 PMC4951292

[35] Lee SE Hwang TS Choi YL Han HS Kim WS Jang MH Prognostic significance of TERT promoter mutations in papillary thyroid carcinomas in a BRAF(V600E) mutation-prevalent population. Thyroid. (2016) 26(7):901–10. 10.1089/thy.2015.0488 27184112

[36] Liu R Bishop J Zhu G Zhang T Ladenson PW Xing M. Mortality risk stratification by combining BRAF V600E and TERT promoter mutations in papillary thyroid cancer: genetic duet of BRAF and TERT promoter mutations in thyroid cancer mortality. JAMA Oncol. (2016) 3(2):202–208. 10.1001/jamaoncol.2016.3288. 27581851

[37] Liu R Xing M. TERT promoter mutations in thyroid cancer. Endocr Relat Cancer. (2016) 23(3):R143–55. 10.1530/ERC-15-0533 26733501 PMC4750651

[38] Kong J Di C Piao J Sun J Han L Chen L Ezrin contributes to cervical cancer progression through induction of epithelial-mesenchymal transition. Oncotarget. (2016) 7(15):19631–42. 10.18632/oncotarget.7779. 26933912 PMC4991407

[39] Melo M da Rocha AG Vinagre J Sobrinho-Simoes M Soares P. Coexistence of TERT promoter and BRAF mutations in papillary thyroid carcinoma: added value in patient prognosis? J Clin Oncol. (2015) 33(6):667–8. 10.1200/JCO.2014.59.4614 25605839

[40] Vinagre J Pinto V Celestino R Reis M Pópulo H Boaventura P Telomerase promoter mutations in cancer: an emerging molecular biomarker? Virchows Arch. (2014) 465(2):119–33. 10.1007/s00428-014-1608-4 25048572

[41] Liu SG Ma L Cen QH Huang JS Zhang JX Zhang JJ. Association of genetic polymorphisms in TERT-CLPTM1L with lung cancer in a Chinese population. Genet Mol Res. (2015) 14(2):4469–76. 10.4238/2015.May.4.4 25966219

[42] Bae E Lee S Kang B Lee E Choi Y Kang H Replication of results of genome-wide association studies on lung cancer susceptibility loci in a Korean population. Respirology. (2012) 17(4):699–706. 10.1111/j.1440-1843.2012.02165.x 22404340

[43] Simon M Hosking F Marie Y Gousias K Boisselier B Carpentier C Genetic risk profiles identify different molecular etiologies for glioma. Clin Cancer Res. (2010) 16(21):5252–9. 10.1158/1078-0432.CCR-10-1502 20847058 PMC2989876

[44] Choi B Yoon J Kim O Choi W Nam S Lee J Influence of the hTERT rs2736100 polymorphism on telomere length in gastric cancer. World J Gastroenterol. (2015) 21(31):9328–36. 10.3748/wjg.v21.i31.9328 26309358 PMC4541384

[45] Chen XF Cai S Chen QG Ni ZH Tang JH Xu DW Multiple variants of TERT and CLPTM1L constitute risk factors for lung adenocarcinoma. Genet Mol Res. (2012) 11(1):370–8. 10.4238/2012.February.16.2 22370939

[46] Myung JK Kwak BK Lim JA Lee MC Kim MJ. TERT promoter mutations and tumor persistence/recurrence in papillary thyroid cancer. Cancer Res Treat. (2016) 48(3):942–7. 10.4143/crt.2015.362 26727717 PMC4946362

[47] Gong L Xu Y Hu YQ Ding QJ Yi CH Huang W hTERT gene polymorphism correlates with the risk and the prognosis of thyroid cancer. Cancer Biomark. (2016) 17(2):195–204. 10.3233/CBM-160631 27472887 PMC13020490

[48] Yin DT Yu K Lu RQ Li X Xu J Lei M Clinicopathological significance of TERT promoter mutation in papillary thyroid carcinomas: a systematic review and meta-analysis. Clin Endocrinol (Oxf). (2016) 85(2):299–305. 10.1111/cen.13017 26732020

